# A Retrospective Study on Wilson Osteotomy with Intramedullary Locking Plate for Failed Hallux Valgus Correction: Insights from a Single-Surgeon Experience

**DOI:** 10.3390/life15101592

**Published:** 2025-10-12

**Authors:** Yi Ping Wei, Meng Chen Kuo, Yi Jiun Chou

**Affiliations:** 1 Department of Orthopedics, Kaohsiung Veterans General Hospital, Kaohsiung 813414, Taiwan; weiy8242@gmail.com (Y.P.W.); winston695@gmail.com (M.C.K.); 2 Institute of Biomedical Sciences, National Sun Yat-sen University, Kaohsiung 518107, Taiwan; 3 Department of Occupational Therapy, Shu-Zen Junior College of Medicine and Management, Kaohsiung 813414, Taiwan; 4 College of Medicine, National Sun Yat-sen University, Kaohsiung 518107, Taiwan; 5 Office of R&D with International Affairs, Tajen University, Pingtung 900391, Taiwan

**Keywords:** bunion, foot, hallux valgus, Wilson osteotomy, subcapital osteotomy, hallux valgus angle, intermetatarsal angle, distal metatarsal articular angle, revision surgery

## Abstract

**Background/Objective**: The recurrence of hallux valgus (HV) after primary surgical correction remains a clinical challenge, often requiring combined approaches to address both bony realignment and soft tissue imbalance. While locking plates have shown some biomechanical advantages in HV correction, evidence regarding their application in revision procedures is limited. This study presents a retrospective single-surgeon experience with a small cohort, aiming to describe radiographic and functional outcomes and to share practical insights rather than provide definitive conclusions. **Methods**: In this retrospective case series, patients undergoing revision surgery for failed HV correction over the past ten years at a single tertiary institution were analyzed. Radiographic parameters—hallux valgus angle (HVA), intermetatarsal angle (IMA), distal metatarsal articular angle (DMAA), and sesamoid position—were assessed. Functional outcomes included the American Orthopaedic Foot and Ankle Society (AOFAS) score and the Visual Analog Scale for pain. Surgical strategies were tailored according to recurrence mechanisms, and some cases involved Wilson osteotomy with intramedullary plate fixation. The Mann–Whitney U test and the Wilcoxon signed-rank test were applied to assess efficacy. **Results**: A total of 11 feet treated by one surgeon were included. Both soft tissue procedures and combined osteotomy with intramedullary plate fixation led to statistically significant but preliminary improvements in HVA, IMA, DMAA, and sesamoid alignment. Functional scores improved, and the complication rate was within the range reported in the previous literature. **Conclusions**: This retrospective single-surgeon study with a limited sample size suggests that Wilson osteotomy combined with intramedullary plate fixation may represent a joint-preserving and biomechanically supportive option for recurrent HV, particularly in cases with large DMAAs and severe sesamoid displacement. However, the findings should be interpreted cautiously given the small cohort, retrospective design, and absence of multi-angle radiographic visualization. The results highlight a potential approach in specific clinical settings rather than a definitive solution. Larger, prospective, multi-center studies are required to confirm long-term utility.

## 1. Introduction

Recurrence of hallux valgus (HV) following surgical correction remains a persistent and challenging clinical issue, with reported failure rates as high as 50% in some series [1]. Among these, recurrent deformity is the most prevalent cause of failure, occurring in approximately 3–16% of cases [1,2]. Revision surgery is particularly demanding when the index procedure involves a metatarsal osteotomy, as insufficient residual bone stock can complicate bony realignment [3,4]. In contrast, when the primary correction relies solely on soft tissue balancing, revision is often hindered by dense postoperative adhesions around the first metatarsophalangeal joint [5,6]. While mild deformities may respond to renewed soft tissue procedures alone, more complex recurrences frequently require a comprehensive revision approach that addresses both osseous and soft tissue pathology to restore joint congruity and biomechanical balance [4]. In this context, HV is increasingly recognized as a three-dimensional deformity, and surgical correction may involve not only lateral translation of the metatarsal head to correct the HVA and the IMA but also coronal and axial plane rotation to correct the DMAA and pronation deformity [7]. Ultimately, the success of revision surgery hinges on the accurate identification of the underlying cause of failure and a tailored surgical strategy based on individual anatomical and biomechanical considerations.

Despite the clinical importance of this issue, the body of literature dedicated to revision HV surgery remains limited. Most existing studies provide general descriptions of techniques or small case series, offering limited guidance on the selection of surgical procedures, choice of fixation, and expected radiographic or functional outcomes [1]. In recent years, several short- to mid-term studies have explored the use of intramedullary or locking plates for primary hallux valgus correction [7,8,9,10]. The authors of these studies employed a variety of osteotomy techniques—including Lindgren, Bösch, and transverse or oblique subcapital osteotomies—with different plating systems, such as Endolog and titanium mini locking plates [7,8,9,10]. Across these reports, sample sizes ranged from 10 to 194 feet, with follow-up periods from 3 to 97 months. Most studies demonstrated significant postoperative improvements in hallux valgus angle (HVA), intermetatarsal angle (IMA), and distal metatarsal articular angle (DMAA), with postoperative HVA values generally reduced to below 15°. Functional outcomes, when reported, showed favorable AOFAS scores, typically in the 69–90 range. Complication rates were generally low, though some studies noted recurrence, hardware-related irritation, or superficial infections. Of particular interest, one 2024 study using a titanium mini locking plate reported a mean first-metatarsal shortening of 1.89 ± 0.9 mm, which was not associated with metatarsalgia or adverse outcomes [10]. While these studies demonstrate that plate fixation can yield favorable radiographic and functional outcomes in primary hallux valgus correction, their applicability to revision cases remains uncertain, as most reports have focused exclusively on primary procedures [7,8,9,10]. Evidence specifically addressing the use of locking plates in revision settings is limited, with only a few studies available—often with small sample sizes and lacking standardized outcome measures [11]. This underscores the need for further research to evaluate the effectiveness and technical considerations of plate fixation in the context of recurrent deformity. Notably, biomechanical studies suggest that locking plates may confer distinct advantages in revision settings by offering greater stability during the gait cycle, particularly in the push-off phase, where a direct abduction force is applied to the metatarsal head [12]. This mechanical superiority may translate into improved postoperative outcomes in cases where previous fixation failed.

Considering these gaps, the present study aims to provide preliminary observations on HV revision surgery using locking plates. Drawing on a decade of institutional experience, we report a small retrospective single-surgeon series, focusing on postoperative radiographic and functional outcomes in patients who underwent revision after failed primary HV correction. Rather than offering definitive conclusions, this study is intended to share practical insights that may assist in preoperative evaluation and surgical planning, and to highlight technical considerations that appeared to be associated with favorable outcomes in this limited cohort. These findings should be interpreted cautiously, but may still offer a useful reference for clinicians working in settings where evidence remains scarce.

## 2. Materials and Methods

### 2.1. Patient Selection and Data Collection

Following approval by the institutional research ethics board (KSVGH24-CT3-23), a retrospective review of the institutional surgical database was conducted to identify patients aged 20 years or older who underwent revision surgery for symptomatic recurrence of hallux valgus (HV) with postoperative follow-up at our hospital over the past ten years.

Patients were included based on the following criteria:

(1) Radiographic evidence of recurrent HV deformity, defined as a hallux valgus angle (HVA) greater than 20 degrees;
(2) Persistent functional impairment or pain affecting daily activities;
(3) Documented failure of conservative treatment, such as orthotic use or physical therapy.

Patients with advanced osteoarthritis of the first metatarsophalangeal joint or active infection were excluded.

A thorough review of medical records and radiographs was conducted to collect data on demographic characteristics, operative details, preoperative and postoperative radiographic parameters, and surgical complications.

The radiographic parameters assessed were HVA, IMA, DMAA, and medial sesamoid grades, with the measurement methods being based on previously published techniques [13,14]. Functional outcomes were evaluated using the American Orthopaedic Foot and Ankle Society (AOFAS) Hallux scale, the Grading Hallux Metatarsophalangeal–Interphalangeal scale [15], and the Visual Analog Scale (VAS) for pain assessment. Follow-up assessments were conducted either in the outpatient clinic or via telephone interviews. The follow-up period was defined as the interval from the date of surgery to the most recent outpatient visit or telephone evaluation. Weight-bearing anteroposterior radiographs were obtained at four time points: preoperatively (prior to revision surgery), one month postoperatively, three months postoperatively, and at the final follow-up. The position of the medial sesamoid was graded according to the classification described by Hardy and Clapham [16]: grades I to III were considered reductions, while grades IV to VII were classified as non-reductions [16]. Postoperative recurrence was defined as an HVA ≥ 20 degrees at any point during the follow-up period [17].

All surgeries were performed at the same institution by a single senior surgeon with over 20 years of experience in orthopedic foot and ankle surgery. Data collection was conducted independently and in duplicate by two authors, both of whom had completed more than six years of orthopedic training. All patient data were de-identified prior to analysis.

A standardized postoperative protocol was applied to all patients. This included an initial 4-week period of non-weight bearing, followed by a 4-week phase of progressive weight bearing using a forefoot offloading shoe.

### 2.2. Statistical Analysis

Data analysis was conducted based on data type and distribution by using descriptive statistical methods. All statistical analyses were performed using SPSS Statistics version 20 (IBM Corp., Armonk, NY, USA), with a *p*-value of <0.05 being considered statistically significant. Continuous variables are presented as the mean ± standard deviation (SD). The Wilcoxon signed-rank test was used to evaluate the effectiveness of the surgical procedure by comparing preoperative measurements with those obtained one month postoperatively; to assess the durability of the surgical outcomes, additional Wilcoxon signed-rank tests were conducted by comparing values at one month and three months postoperatively, as well as by comparing values at three months postoperatively and the final follow-up. The Mann–Whitney U test was used for between-group comparisons. Measurement reliability was assessed using intraclass correlation coefficients (ICCs), and interobserver reliability was measured separately twice by 2 independent investigators (M.C.K. and Y.P.W.) and was determined by comparing measurements.

### 2.3. Surgical Strategies

When managing recurrent HV deformity, it is essential to identify the surgical technique used in the initial procedure and determine the underlying reasons for its failure. Based on clinical experience, the most commonly observed contributing factors include the following:

1. An incomplete reduction in sesamoid position, leading to persistent joint misalignment;
2. Failure to correct the DMAA, resulting in the inadequate realignment of the first metatarsus;
3. An unaddressed coronal plane rotation of the first metatarsal, which has been increasingly recognized as a critical factor in recurrence.

A recent study has confirmed that HV is a three-dimensional deformity, and failure to correct coronal plane rotation may compromise the durability of surgical outcomes [7,16]. Therefore, our surgical strategy aims to address both angular deformities and rotational malalignment.

**Figure 1:**
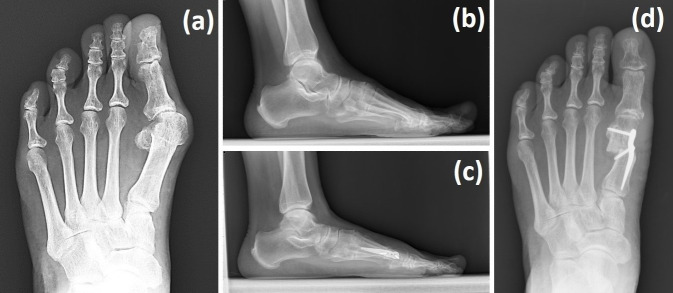
HV revision surgery performed using the Spear plate with Wilson osteotomy combined with a distal soft tissue procedure. The left foot of a 63-year-old male patient with HV recurrence was corrected, as shown in the above weight-bearing radiographs. (**a**,**b**) Preoperative radiographs and (**c**,**d**) 3-month-postoperative radiographs.

At our institution, the senior surgeon employs a targeted step-wise algorithm incorporating the Spear plate (Aplus Biotechnology Company, New Taipei City, Taiwan), Wilson osteotomy, and/or distal soft tissue balancing depending on preoperative findings (Figure 1).

In particular, patients with sesamoid non-reduction or a DMAA ≥ 15° and without tarsometatarsal (TMT) joint hypermobility or degenerative changes undergo a Wilson osteotomy with correction in both the transverse and frontal planes, combined with distal soft tissue release and Spear plate fixation.

Patients with a DMAA < 15° and a reducible metatarsophalangeal (MTP) joint (in neutral and 15° hallux dorsiflexion) and without evidence of TMT joint instability are treated with a distal soft tissue procedure alone.

This algorithm allows for the individualized correction of HV deformities, addressing both angular and rotational components while avoiding unnecessary osteotomies in milder cases.

### 2.4. Surgical Technique

Revision surgery for recurrent hallux valgus was performed using a standardized approach centered on the application of the Spear locking plate. The procedure included a bunionectomy, capsular release, and a linear subcapital osteotomy performed approximately 2 cm proximal to the metatarsal head. Dorsal and plantar periosteal stripping was carefully executed to avoid injury to the dorsomedial cutaneous nerve. The osteotomy was performed at an angle of approximately 15 degrees to a line perpendicular to the metatarsal shaft, with the saw blade being directed laterally toward the midshaft of the fifth metatarsal to minimize the risk of undesirable lengthening or shortening, which can contribute to transfer metatarsalgia.

Following the osteotomy, the metatarsal head was translated laterally and fixed using the Spear intramedullary locking plate. Special attention was paid to bone alignment and screw orientation to ensure stable fixation.

### 2.5. Technical Considerations in Revision Settings

In revision hallux valgus surgery, technical precision is essential due to altered anatomy, limited distal bone stock, and higher recurrence risk. Based on our limited single-surgeon experience, several intraoperative strategies were applied with the intent of improving correction accuracy and construct stability, although these should be interpreted as practical observations rather than definitive recommendations.

#### 2.5.1. Sesamoid Reduction and Coronal Plane Alignment

Particular emphasis was placed on achieving adequate release of the metatarso–sesamoid ligament complex during lateral soft tissue release. Following this step, the reducibility of the first MTP joint to 30–60 degrees of varus stress was assessed, serving as a clinical indicator of sufficient lateral release and joint mobility. To further facilitate sesamoid reduction, controlled medial traction was applied using a Kelly clamp to gently retract the medial capsule and adductor tendon complex. Intraoperative fluoroscopy was utilized to evaluate the position of the sesamoids and the alignment of the metatarsal head. While axial imaging (e.g., weight-bearing CT) offers ideal visualization, previous studies have suggested that dorsoplantar intraoperative fluoroscopy—when combined with the careful assessment of the metatarsal head contour and shape—can reliably reflect rotational alignment and sesamoid position [18,19]. A symmetric and rounded metatarsal head without torsional distortion provides indirect evidence of appropriate coronal plane correction. Once satisfactory reduction is confirmed, medial capsular plication is performed to maintain the corrected alignment. This step-wise approach addresses both the angular and rotational components of the deformity.

#### 2.5.2. Fixation Stability in the Setting of Limited Bone Stock

**Figure 2:**
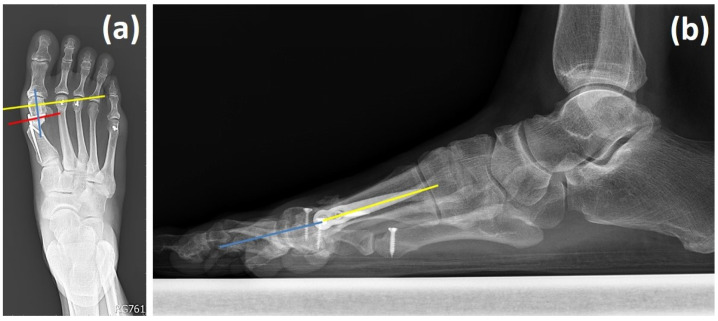
Two angles to be noted when using the Spear plate. (**a**) Anteroposterior view of the distal screw insertion angle between the distal screw on the plate (red line) and the line perpendicular to the axis of the distal fragment (yellow line); the axis of the distal fragment (blue line). (**b**) Lateral view of the lateral angulation angle between the axis of the metatarsal head (blue line) and the axis of the proximal metatarsal shaft (yellow line).

Due to the limited distal bone stock frequently encountered in revision procedures, several fixation strategies were applied to improve construct integrity:

1. Distal screw insertion angle: The trajectory of the distal locking screw was carefully adjusted relative to the perpendicular axis of the distal bone fragment to minimize the risk of sagittal plane rotation (Figure 2a). A slight angulation, rather than a strictly perpendicular orientation, was used to improve rotational stability.
2. Lateral angulation monitoring: Intraoperative lateral radiographs were examined to ensure proper alignment between the distal metatarsal head and the proximal shaft, allowing for the detection and correction of any angular deformity (Figure 2b).
3. Preservation of metatarsal length: Postoperative changes in metatarsal length were monitored, as inadvertent lengthening may increase the risk of complications such as metatarsalgia, nonunion, or implant failure. To reduce this risk, periosteal disruption was minimized during osteotomy and plate placement.

Overall, these technical considerations represent practical measures employed in this limited series to enhance fixation stability and alignment in the revision setting. They should be regarded as preliminary observations that may inform surgical planning, but confirmation in larger and prospective studies is needed.

## 3. Results

A total of 11 feet in nine patients were operated on by the same surgeon in the same hospital over the past ten years. The mean follow-up was 27.36 months (range, 9–55 months). The average age at the time of surgery was 65.55 (range 51–77) years (Table 1).

Table 2 shows that the ICC values for this study ranged from 0.851 to 0.996 (*p* < 0.001), with very high reliability.

The mean preoperative AOFAS scores were 55.25 ± 3.30 in the group who underwent the distal soft tissue procedure alone and 61.50 ± 12.96 in the group who underwent Wilson osteotomy with Spear plate fixation, with no statistically significant difference between the two groups (*p* = 0.114). At the final follow-up, the postoperative AOFAS scores improved to 65.75 ± 4.57 and 80.43 ± 3.55, respectively, i.e., increasing by 10.50 ± 3.11 in the soft tissue group and 19.17 ± 10.83 in the Spear plate group, respectively, without reaching statistical significance (*p* = 0.067).

Regarding the pain outcomes, the preoperative VAS scores were 6.00 ± 1.16 in the soft tissue group and 5.50 ± 0.55 in the Spear plate group (*p* = 0.610). At the final follow-up, the postoperative VAS scores improved to 2.25 ± 0.50 and 1.33 ± 0.52, respectively. Although the Spear plate group appeared to show greater pain reduction, the between-group difference did not reach statistical significance, suggesting that the observed trend may not be generalizable (*p* = 0.067).

### 3.1. Postoperative Changes in HVA, IMA, and DMAA: A Comparative Analysis of Soft Tissue Plication Alone Versus Soft Tissue Plication with Bone Osteotomy

Based on the Wilcoxon signed-rank test, we found that the HVA significantly improved in the group who underwent Wilson osteotomy with Spear plate fixation from the preoperative period to one month postoperatively (*p* = 0.018). In contrast, no statistically significant improvement in the HVA was observed in the group that underwent the distal soft tissue procedure alone (*p* = 0.068). At three months postoperatively and at the final follow-up, the HVA remained stable without significant deterioration in either group (all *p* > 0.05).

Due to the nature of the procedure, the distal soft tissue procedure alone had a minimal effect on correcting the IMA and the DMAA, with no statistically significant changes being observed across all time points. However, in the group who underwent the Wilson osteotomy with Spear plate fixation, both the IMA and the DMAA significantly improved one month postoperatively (IMA: *p* = 0.018; DMAA: *p* = 0.018). Notably, these angles continued to show mild but statistically significant changes between one and three months postoperatively (IMA: *p* = 0.046; DMAA: *p* = 0.028), although the magnitude of these changes was minimal (IMA: from 5.20° to 6.49°; DMAA: from 2.13° to 4.50°) and may be of limited clinical relevance. Beyond three months, both the IMA and the DMAA tended to stabilize, with no further statistically significant differences being observed (IMA: *p* = 0.785; DMAA: *p* = 0.528).

### 3.2. Impact of Surgical Techniques on Sesamoid Position

From Table 1 and Table 3, the group that underwent Wilson osteotomy with Spear plate fixation shows a good corrective effect on sesamoid grades. In the group that underwent the soft tissue procedure alone, although the mean sesamoid grade was reduced by 0.5 postoperatively, the mean postoperative sesamoid grade remained at 4.0, which is still considered a medial sesamoid non-reduction.

However, because the choice of revision surgery was influenced by preoperative sesamoid grade (with patients presenting with higher grades more likely to undergo a combined osteotomy and soft tissue procedure), objective comparisons of effectiveness between the two techniques are not possible. Nevertheless, among patients with a high preoperative sesamoid grade, a combined osteotomy and soft tissue procedure appeared to yield more favorable outcomes; however, this observation should be interpreted cautiously in light of the study’s small sample size and limited scope.

## 4. Discussion

The findings of this study indicate that the DMAA may be partially corrected in patients who underwent Wilson osteotomy with Spear plate fixation. Although a statistically significant worsening trend was noted within the first three months postoperatively, the actual magnitude of change was small and may have limited clinical relevance. Beyond this early period, the DMAA appeared to stabilize without further statistically significant deterioration. These results should be interpreted cautiously, as they are based on a small retrospective series, and further studies with larger cohorts and longer follow-up are needed to clarify the durability and clinical significance of these radiographic changes. When managing recurrent HV deformity, it is essential to identify the surgical technique used in the initial procedure and determine the reasons for its failure [1]. Patient-related factors that may contribute to recurrence include smoking, which can lead to wound infection or nonunion, as well as underlying conditions such as rheumatoid arthritis, hypothyroidism, gout, diabetic neuropathy, hereditary neuromuscular disorders, Parkinson’s disease, and cerebral palsy [1]. Surgeon-related factors include the selection of an inappropriate surgical approach and lack of a comprehensive postoperative management plan [1,20,21]. According to the established surgical strategy, the senior surgeon at our hospital arranged the surgical procedure based on preoperative sesamoid grades, the DMAA, and whether the MTP joint was reducible on physical examination. The surgical options included the soft tissue procedure alone and Wilson osteotomy with Spear plate fixation. Most cases achieved good treatment outcomes during the follow-up period. Only one patient who underwent the soft tissue procedure alone experienced HV recurrence. No patients required hardware removal surgery during the follow-up period. Notably, among the 11 cases, 1 patient requested the use of a Spear plate for HV revision surgery on the contralateral foot due to a good experience with a previous revision procedure using the same implant, despite a preoperative DMAA of less than 15 degrees (the preoperative DMAA for this case was 12.9 degrees). Another patient had high early postoperative activity demands, and after discussion, a Spear plate was also used for fixation despite a preoperative DMAA below 15 degrees (the preoperative DMAA for this case was 14.8 degrees).

Our case series consists exclusively of patients who underwent revision surgery. To the best of our knowledge, studies evaluating the use of intramedullary plates for HV re-correction in revision procedures are scarce [11]. While our findings suggest that performing revision surgery with an HV-specific intramedullary plate may provide certain biomechanical advantages and yield acceptable postoperative outcomes in patients with a high preoperative sesamoid grade and a large DMAA, these results should be regarded as preliminary rather than definitive evidence of superiority. Compared with a 2025 study on the use of intramedullary plates in revision hallux valgus correction, our study demonstrated a comparable complication rate, while the preoperative-to-postoperative improvements in HVA, IMA, and DMAA were largely consistent with their findings, supporting the potential reproducibility of this surgical technique but not establishing its clear advantage (Table 4) [11].

Two implant-related complications were observed in our cohort, both occurring around the third postoperative month. One case involved distal screw breakage, likely due to cyclic bending stress in an unstable distal fragment. The current Spear plate design utilizes a single, horizontally oriented distal locking screw, which may act as a pivot point rather than providing adequate sagittal plane stability—particularly during early ambulation [22]. The second case involved proximal screw pull-out, most likely attributable to insufficient screw purchase in osteoporotic bone as the patient transitioned to partial weight bearing. These events suggest a potential mismatch between implant design and local bone quality, especially in revision cases. Despite these complications, both patients were successfully managed nonoperatively. Conservative treatment, including extended non-weight-bearing and functional bandaging in a hypercorrected position, led to satisfactory outcomes, and the final AOFAS scores were 87 and 82 at 9- and 13-month follow-up, respectively. While radiographic union was incomplete—showing no clear bony union in one case and partial union in the other—the preserved alignment and absence of pain highlight that implant failure does not necessarily equate to surgical failure, provided that biological and mechanical environments remain favorable. Nevertheless, the clinical significance of these implant-related complications must be recognized, further underscoring that our conclusions should be interpreted with caution.

To reduce the risk of such mechanical failures, several implant design refinements and technical strategies may be considered. From a design perspective, reliance on a single distal fixation point and limited cortical purchase proximally may predispose the construct to instability under repetitive stress [22]. Potential improvements include incorporating two distal fixation points, variable-angle locking options, or longer/multi-planar screw trajectories to enhance fixation strength. In osteoporotic bone, the use of bone graft augmentation or cement augmentation may also improve purchase and reduce early loosening. Beyond implant design, surgical refinements may help mitigate mechanical complications. Supplementing fixation with soft tissue balancing procedures or biologic augmentation could further improve construct durability, particularly in revision cases with compromised bone quality.

Additionally, adopting alternative osteotomy techniques, such as combining a horizontal distal osteotomy with an Akin osteotomy, may help to distribute mechanical stress more evenly, thereby reducing load on the distal screw [23]. Preoperative assessment of bone mineral density and individualized implant selection may also aid in identifying high-risk patients who could benefit from reinforced fixation strategies. Modifying postoperative protocols—including delayed ambulation or extended protected weight bearing—may also be beneficial in cases of severe deformity or limited bone stock.

These observations underscore the need for improved implant design, individualized fixation planning, and tailored postoperative management to optimize outcomes and minimize complications in revision hallux valgus surgery.

This study has several important limitations. First, its retrospective design introduced inherent biases, as preoperative AOFAS scores were obtained from patients’ postoperative recollection rather than contemporaneous assessment, thereby introducing potential recall bias. The small sample size may have led to either overestimation or underestimation of outcomes, and the relatively short follow-up period limited the ability to draw conclusions regarding long-term durability. Nevertheless, although a few patients had follow-up shorter than one year, the minimum follow-up duration exceeded six months in all cases, allowing for a preliminary evaluation of recurrence based on the existing literature [24,25]. Importantly, the absence of a control group further restricted the ability to draw causal inferences or compare this technique with alternative fixation methods.

Regarding radiographic evaluation, the retrospective design limited access to advanced imaging; thus, our analysis was restricted to transverse plane parameters such as HVA, IMA, DMAA, and sesamoid grade. This constraint precluded a comprehensive three-dimensional assessment, although the role of axial and coronal plane imaging (e.g., weight-bearing CT) is increasingly recognized as essential.

All surgeries were performed by a single experienced surgeon, which ensured technical consistency but may also reduce the generalizability of the findings to other institutions or surgeons with different levels of expertise. Additionally, implant-related complications—including distal screw breakage and proximal screw pull-out—were observed, particularly in patients with poor bone quality. Moreover, patient-reported outcomes, such as quality-of-life and activity-level measures, were not systematically collected, further limiting the scope of functional evaluation.

Taken together, the small sample size, retrospective design, absence of a control group, limited statistical power, restricted imaging assessments, and implant-specific complications underscore the need for future prospective, multicenter studies with larger cohorts, standardized outcome measures (e.g., AOFAS score, VAS score, patient-reported outcomes), and longer follow-up.

## 5. Conclusions

In conclusion, this retrospective single-surgeon case series suggests that combining Wilson metatarsal osteotomy with intramedullary locking plate fixation may provide a joint-preserving option for selected patients with recurrent HV, particularly those with large preoperative DMAAs and advanced sesamoid displacement. While the construct offers potential biomechanical advantages, the present findings should be regarded as preliminary and interpreted with caution in view of the small sample size, retrospective design, and relatively short follow-up period. This study does not establish definitive recommendations but offers practical observations that may inform surgical planning in similar clinical settings. Larger, prospective, multi-center studies are required to clarify its long-term efficacy and safety.

## Figures and Tables

**Table 1:** Preoperative and postoperative demographic and radiological characteristics of the patients.

Type of Revision Surgery	Distal Soft Tissue Procedure Alone	Wilson Osteotomy with Spear Plate Fixation
Number of cases	4	7
Age ^a^	65.50 ± 10.97	65.57 ± 7.79
Gender	4 females	7 females
Left/right limb	2L2R	2L4R
Previous surgery	1 scarf and 3 MT1 distal osteotomies	5 bunionectomies and 2 MT1 distal osteotomies
Pre-OP sesamoid grade ^a^	4.50 ± 0.58	5.50 ± 0.84 ^b^
Pre-OP HVA ^a^	23.75 ± 3.82	37.16 ± 9.94
Pre-OP IMA ^a^	10.55 ± 3.17	11.81 ± 2.49
Pre-OP DMAA ^a^	7.20 ± 3.65	20.01 ± 5.48
One-month post-OP HVA ^a^	9.53 ± 2.79	9.49 ± 5.55
One-month post-OP IMA ^a^	9.58 ± 2.42	5.20 ± 4.52
One-month post-OP DMAA ^a^	6.28 ± 2.94	2.13 ± 2.45
Three-month post-OP sesamoid grade ^a^	4.00 ± 0.00	3.17 ± 0.41 ^b^
Three-month post-OP HVA ^a^	12.20 ± 8.65	10.60 ± 6.80
Three-month post-OP IMA ^a^	9.55 ± 2.47	6.49 ± 4.63
Three-month post-OP DMAA ^a^	6.23 ± 2.76	4.50 ± 2.25
HVA at final follow-up ^a^	14.03 ± 9.62 ^c^	7.53 ± 6.70 ^d^
IMA at final follow-up ^a^	9.43 ± 3.05 ^c^	4.87 ± 2.99 ^d^
DMAA at final follow-up ^a^	7.30 ± 3.17 ^c^	4.23 ± 1.57 ^d^
Number of HV recurrence cases	1	0
Pre-OP AOFAS ^a^	55.25 ± 3.30	61.50 ± 12.96 ^e^
Post-OP AOFAS ^a^	65.75 ± 4.57	80.43 ± 3.55
AOFAS score improvement ^a^	10.50 ± 3.11	19.17 ± 10.83 ^e^
Pre-OP VAS	6.00 ± 1.16	5.50 ± 0.55 ^e^
Post-OP VAS	2.25 ± 0.50	1.33 ± 0.52 ^e^

Abbreviations: HVA, hallux valgus angle; IMA, intermetatarsal angle; DMAA, distal metatarsal articular angle; L, left; R, right; AOFAS, American Orthopedic Foot and Ankle Society; MT, metatarsus. ^a^ Mean ± SD. ^b^ One case lacked medial sesamoid bones. ^c^ n = 3; one patient did not complete the final X-ray examination due to personal reasons. ^d^ n = 6; one patient was unable to complete the final X-ray examination due to a severe illness (non-hallux valgus-related disease). ^e^ n = 6; one patient could not be contacted for the retrospective assessment.

**Table 2:** Intraclass correlation coefficients.

	Interobserver Correlation	95% Confidence Interval	*p*-Value
Pre-OP HVA	0.996	0.977–0.999	<0.001
Pre-OP IMA	0.953	0.843–0.987	<0.001
Pre-OP DMAA	0.975	0.911–0.993	<0.001
One-month post-OP HVA	0.985	0.894–0.997	<0.001
One-month post-OP IMA	0.959	0.863–0.989	<0.001
One-month post-OP DMAA	0.888	0.594–0.970	<0.001
Three-month post-OP HVA	0.993	0.974–0.998	<0.001
Three-month post-OP IMA	0.978	0.923–0.994	<0.001
Three-month post-OP DMAA	0.851	0.552–0.957	<0.001
HVA at final follow-up	0.990	0.959–0.998	<0.001
IMA at final follow-up	0.925	0.702–0.983	<0.001
DMAA at final follow-up	0.923	0.675–0.982	<0.001

**Table 3:** Postoperative changes in HVA, IMA, DMAA, and sesamoid grade.

		*p*-Value (Preoperatively vs. One Month Postoperatively)	*p*-Value (One Month Postoperatively vs. Three Months Postoperatively)	*p*-Value (Three Months Postoperatively vs. Final Follow-Up)
Distal soft tissue procedure alone (n = 4)	HVA	0.068 ^a^	0.715 ^a^	0.317 ^a^
	IMA	0.285 ^a^	0.317 ^a^	0.180 ^a^
	DMAA	1.000 ^a^	0.713 ^a^	0.285 ^a^
	Sesamoid grade	0.157 ^a,b^		
Wilson osteotomy with Spear plate fixation (n = 7)	HVA	0.018 ^a^	0.600 ^a^	0.248 ^a^
	IMA	0.018 ^a^	0.046 ^a^	0.785 ^a^
	DMAA	0.018 ^a^	0.028 ^a^	0.528 ^a^
	Sesamoid grade	0.023 ^a,b^		

^a^ Wilcoxon signed-rank test. ^b^ Preoperatively vs. three months postoperatively.

**Table 4:** Tabulated data on addressing recurrent hallux valgus with revision osteotomy and intramedullary plate extracted from the published medical literature.

Study (Year)	Number of Cases	Pre-OP Angles and AOFAS Score ^a^	Revision Technique	Implant	Follow-Up (Months) ^b^	Improvement in Angles and AOFAS Score ^c^	Complications
Pérez-Fernández, A et al. (2025) [11]	30	HVA: 34.0 (25.0–60); IMA: 12.0 (6.0–16.5); PASA: 27.0 (4.0–58.0); AOFAS score: 43.0 (19.0–60.0)	Distal osteotomy	Astrolabe© (Astrolabe Medical, Lisbon, Portugal) or V-Tek® (Zimmer Biomet Spain S.L., Barcelona, Spain)	41.0 (24.0–120.0)	HVA: 19.0; IMA: 7.1; PASA: 14.3; AOFAS score: 39.5	21.9% Clavien-Dindo ≥ IIIa (surgical material loosening: 4; surgical material rejection: 2, M1 head necrosis that required arthrodesis: 1)
Present study	7	HVA: 35.3 (24.4–51.5); IMA: 11 (8.3–16.0); DMAA: 20.8 (12.9–28.2) AOFAS score: 65.5 (37–73)	Distal osteotomy	Spear plate (Aplus Biotechnology Company, New Taipei City, Taiwan)	22.0 (9.0–34.0)	HVA: 27.5; IMA: 5.9; DMAA: 19.7 AOFAS score: 17	Distal screw break 3-month postoperatively: 1; proximal screw pull-out 3-month postoperatively: 1

^a^ Median (range); for consistency, the data in this table are presented in the median (range) format, following the approach used in the previous literature. ^b^ Median months (range). ^c^ Median of the differences.

## Data Availability

The datasets generated and analyzed during the current study are not publicly available due to institutional privacy regulations, but are available from the corresponding author upon reasonable request.

## Abbreviations

HV	hallux valgus
HVA	hallux valgus angle
IMA	intermetatarsal angle
DMAA	distal metatarsal articular angle
MTP	metatarsophalangeal
SD	standard deviation
AOFAS	American Orthopedic Foot and Ankle Society
